# Supplementary material for: How Alkali Metal Cations Affect the Structure and Reactivity of the Hydrated Dielectron
Source: J Phys Chem B. 2026 Oct 23;130(44):11875–86. doi: 10.1021/acs.jpcb.6c04903 (PMC13746786; doi:10.1021/acs.jpcb.6c04903)
Supplement: Supplementary file 1 [file jp6c04903_si_001.pdf]

Supplementary Information: How Alkali Metal Cations Affect the Structure and Reactivity of the Hydrated Dielectron

Tatiana Nemirovich,[1] Pavel Jungwirth,[1] and Ondrej Marsalek[2,*]
[1] *Institute of Organic Chemistry and Biochemistry of the Czech Academy of Sciences, Flemingovo nám. 2, 166 10 Prague 6, Czech Republic*
[2] *Charles University, Faculty of Mathematics and Physics, Ke Karlovu 3, 121 16 Prague 2, Czech Republic*

(Dated: 10 September 2026)

S1. RADIAL DISTRIBUTION FUNCTIONS OF EACH SYSTEM

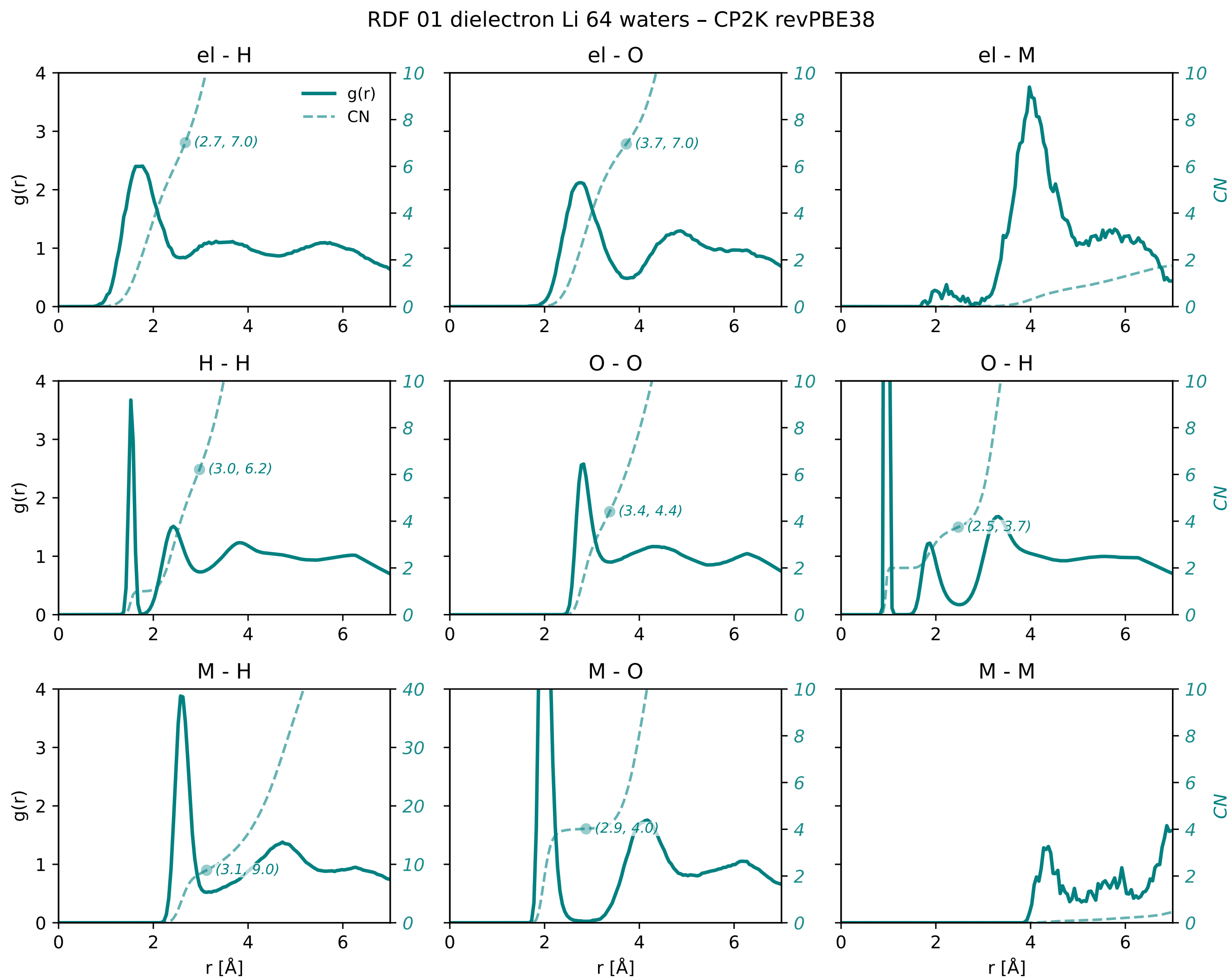

FIG. S1. RDFs and integrated coordination numbers of the system with Li cations. The marker shows the position and value of coordination number in the first minimum of the RDF.

*) Electronic mail: ondrej.marsalek@matfyz.cuni.cz

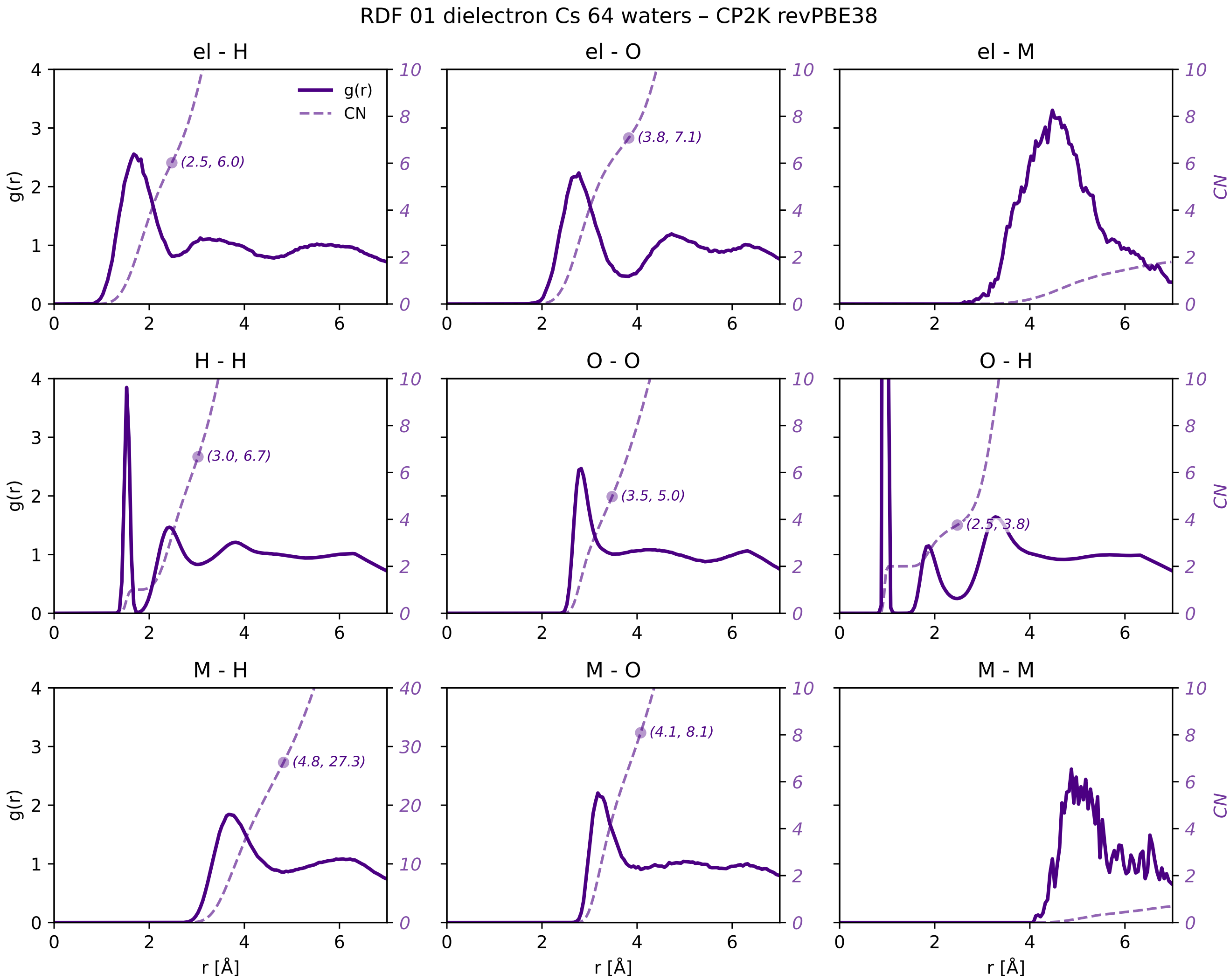

FIG. S2. RDFs and integrated coordination numbers of the system with Cs cations. The marker shows the position and value of coordination number in the first minimum of the RDF.

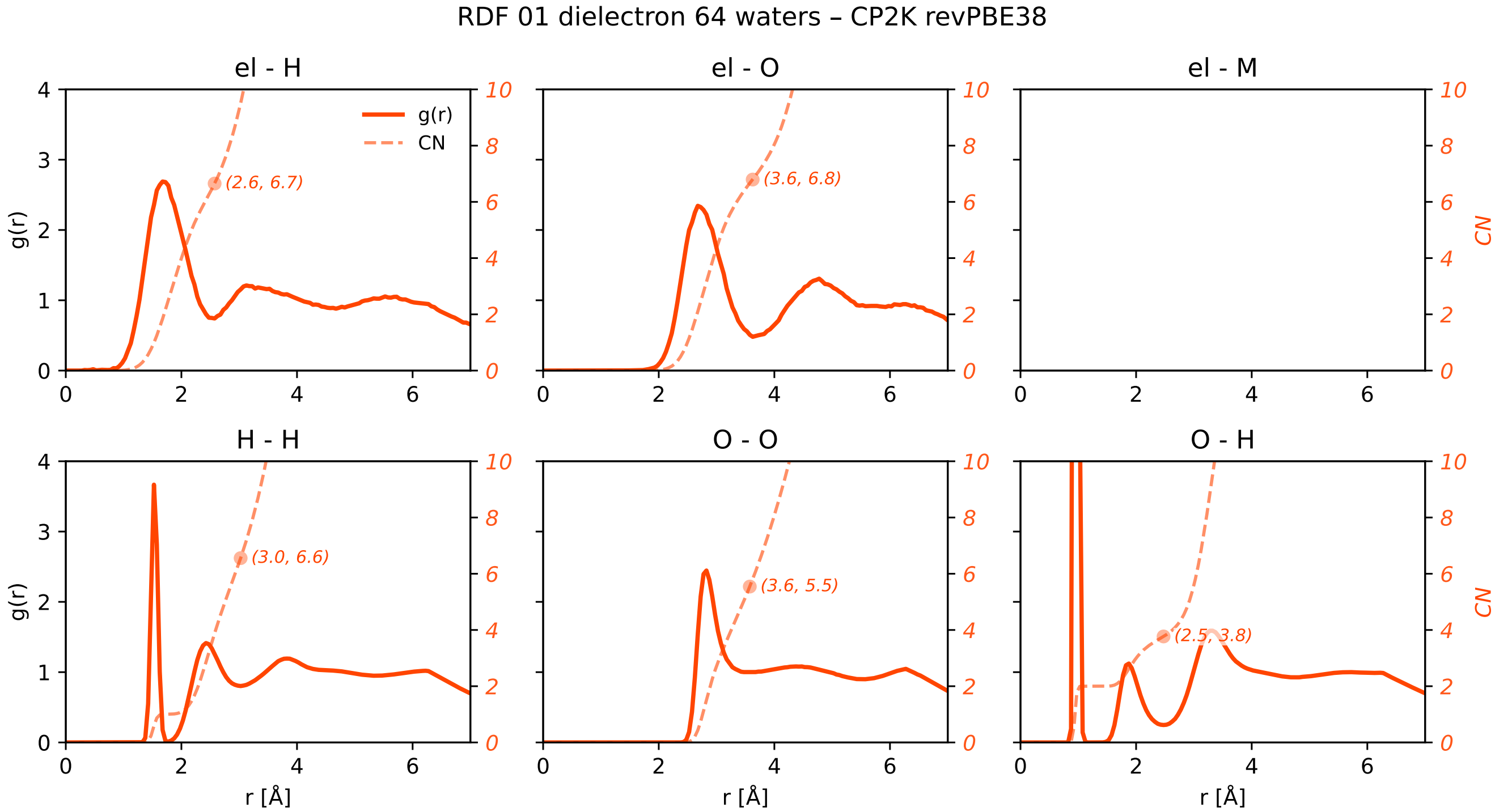

FIG. S3. RDFs and integrated coordination numbers of the system with no cations. The marker shows the position and value of coordination number in the first minimum of the RDF.

S2. RADIAL DISTRIBUTION FUNCTIONS OF WATERS CLOSE TO DIELECTRON

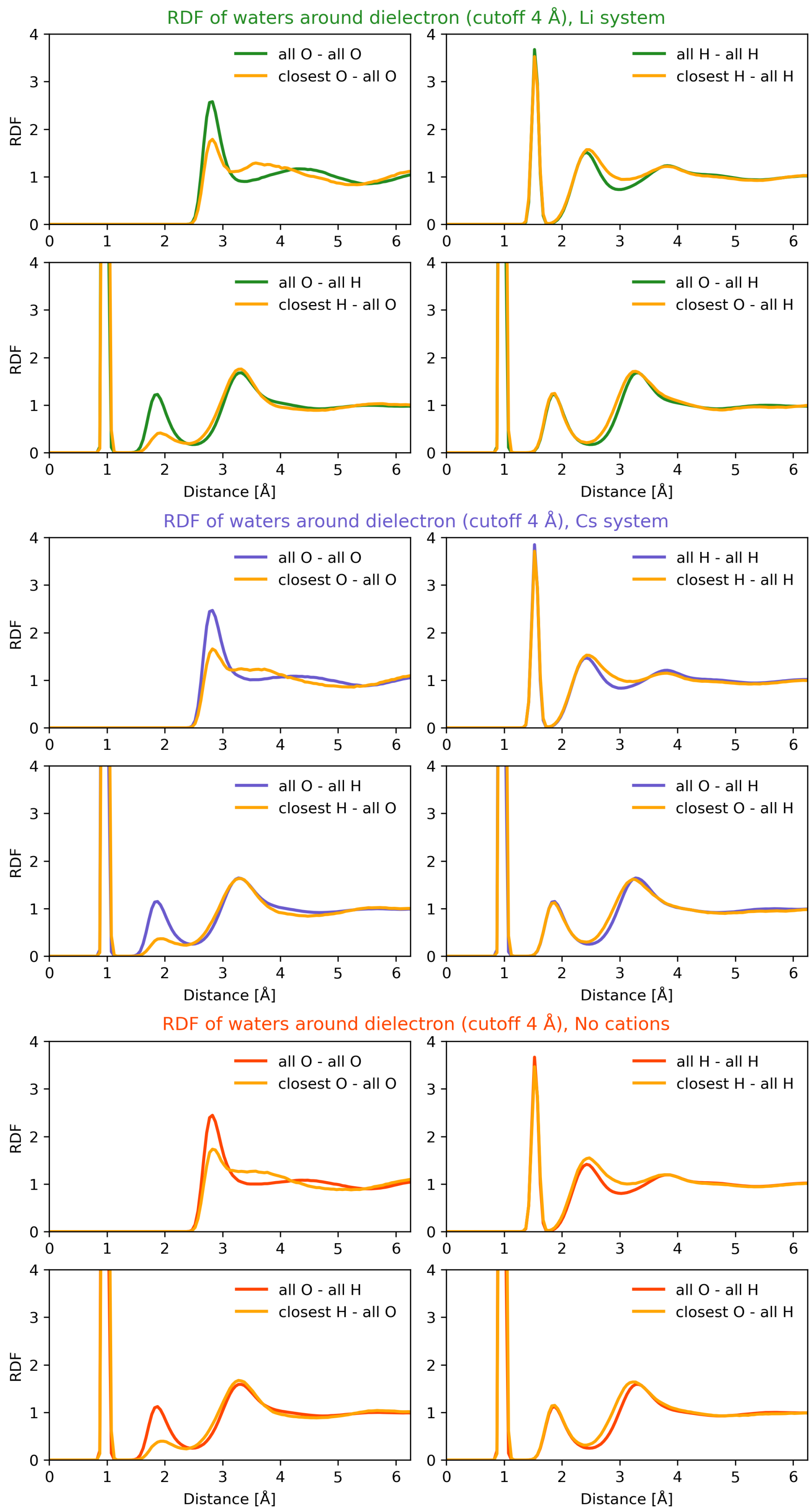

FIG. S4. Water–water RDFs for molecules closest to the dielectron (within a 4 Å cutoff) and the remaining solvent molecules.

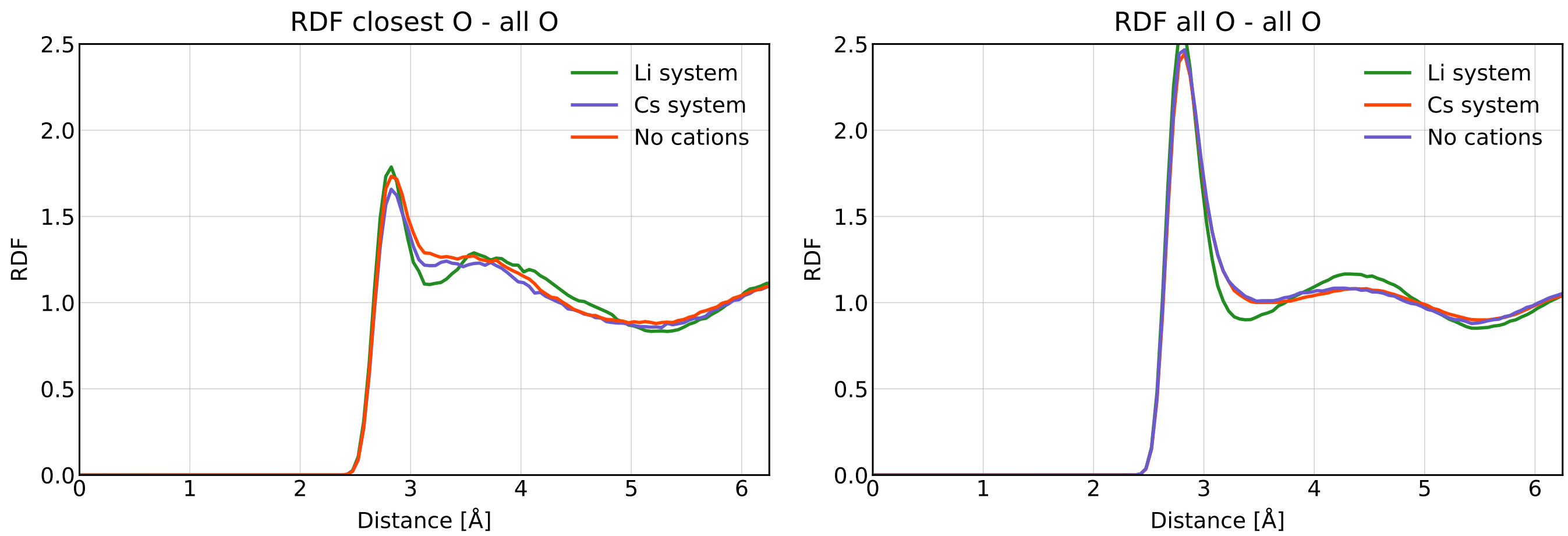

FIG. S5. Left: oxygen–oxygen RDFs for oxygen atoms closest to the dielectron (within a 4 Å cutoff) and the remaining oxygen molecules. Right: overall oxygen–oxygen RDFs containing all possible oxygen pairs for comparison (the same data as presented in Figure 2).

S3. TIME EVOLUTION OF THE GYRATION RADIUS

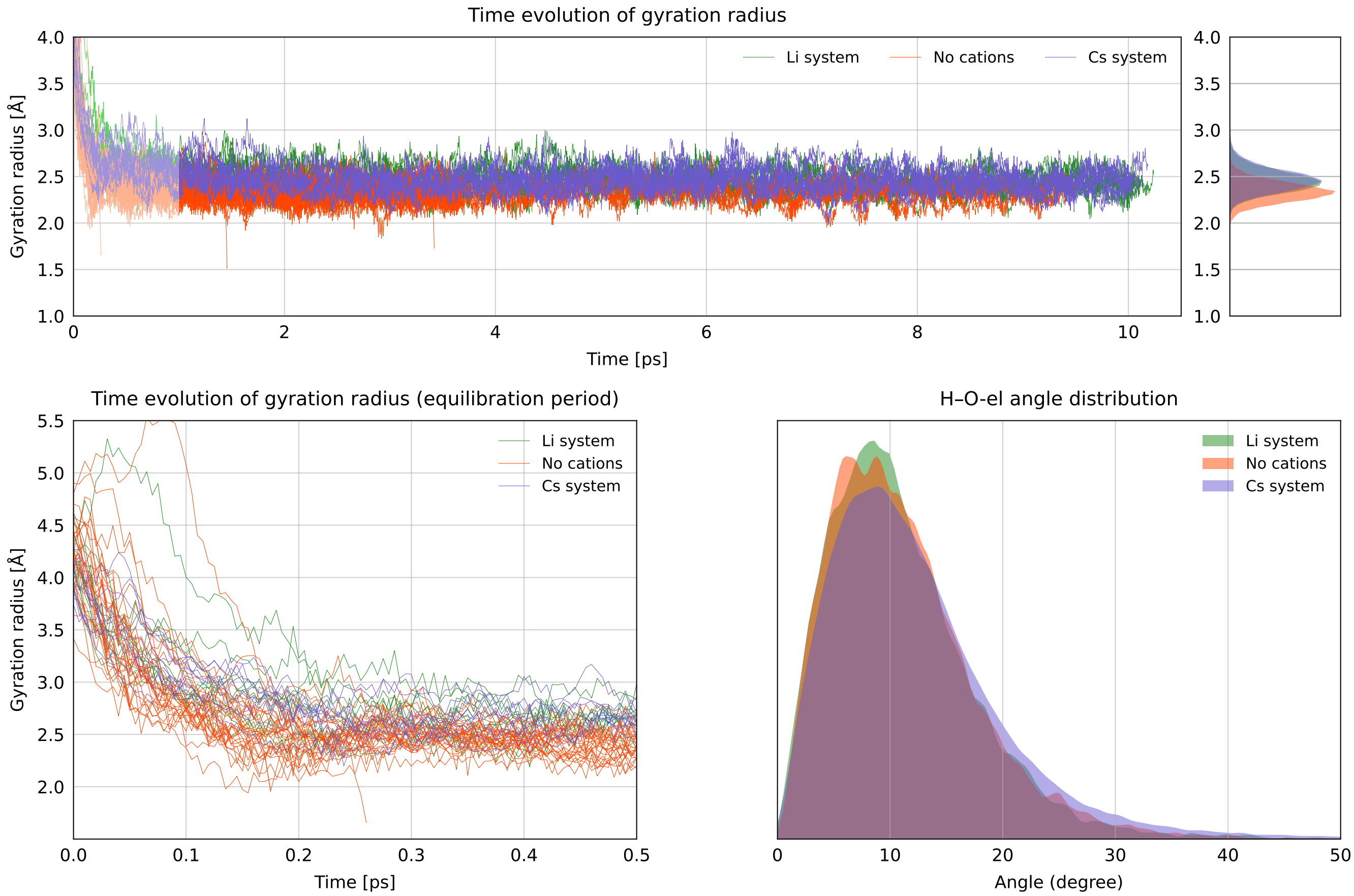

FIG. S6. Top: Time evolution of the gyration radius for each individual trajectory with the equilibration period that is excluded from analysis shown in lighter color. Bottom left: Zoomed-in first 0.5 ps period of all trajectories showing the decease of the gyration radius. Bottom right: Distribution of the H–O–dielectron angles showing that the OH bond is almost parallel to the dielectron–oxygen direction.

S4. DIELECTRON–CATION INTERACTIONS

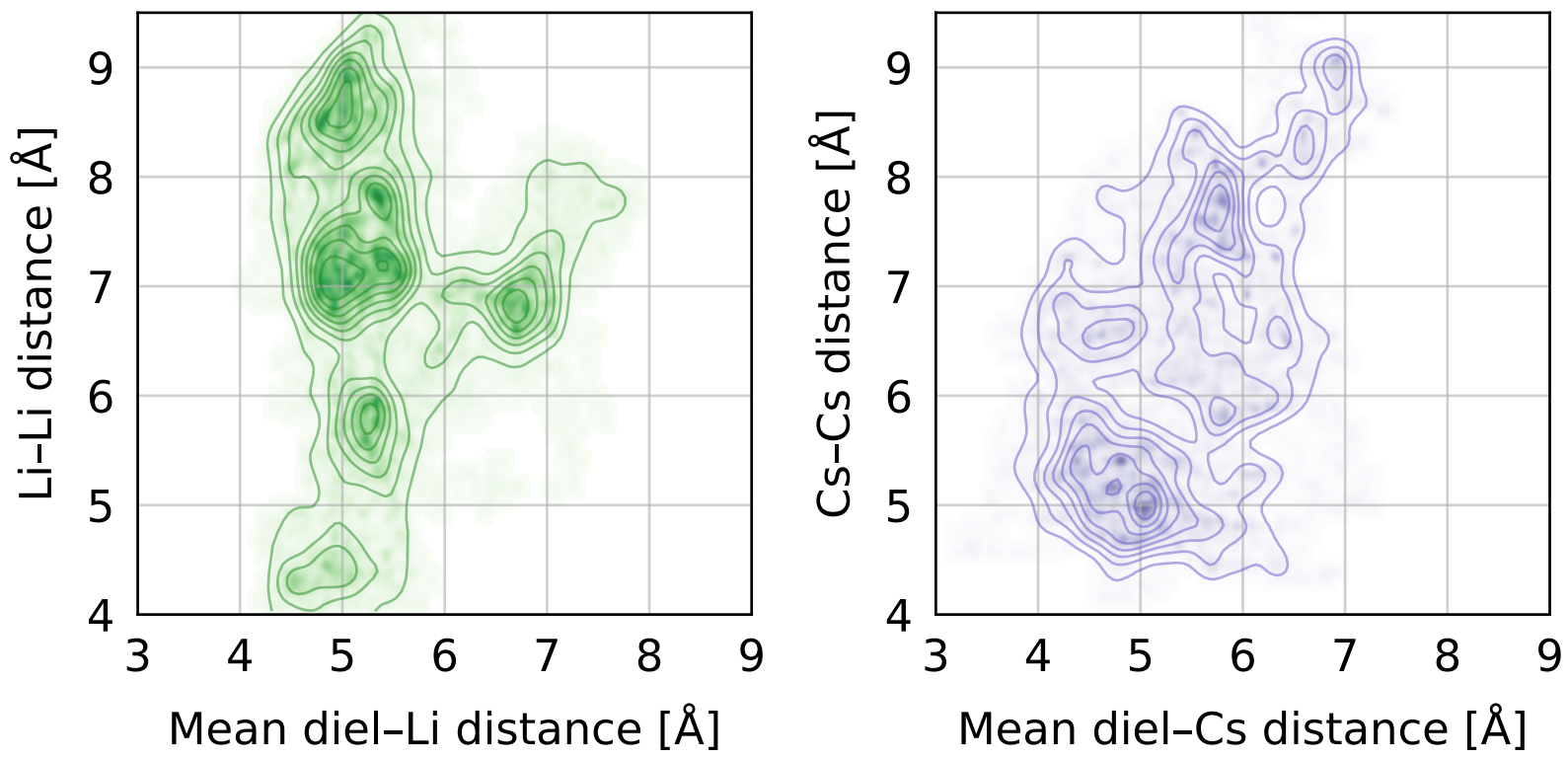

FIG. S7. Two-dimensional probability density of the mean distance between the dielectron center and metal cations and the distance between the two metal cations. Data for Li^+ is shown on the left, while data for Cs^+ is shown on the right.

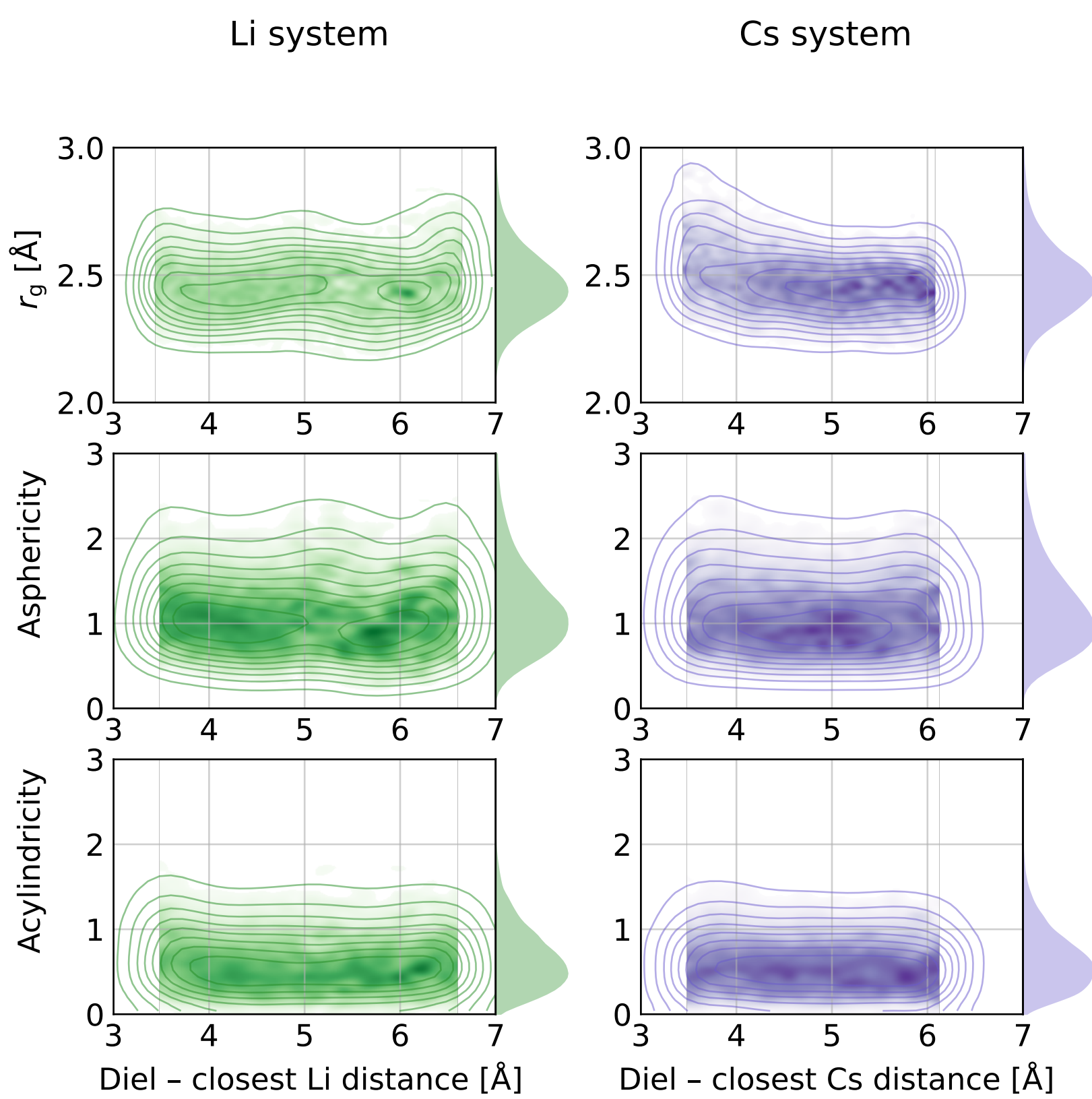

FIG. S8. Correlation between dielectron shape descriptors (r_g, asphericity, and acylindricity) and the distance to the nearest metal cation for Li^+ (left) and Cs^+ (right) normalized separately for every distance.

S5. TWO-DIMENSIONAL DISTRIBUTIONS OF GYRATION RADIUS AND SHAPE ANISOTROPY

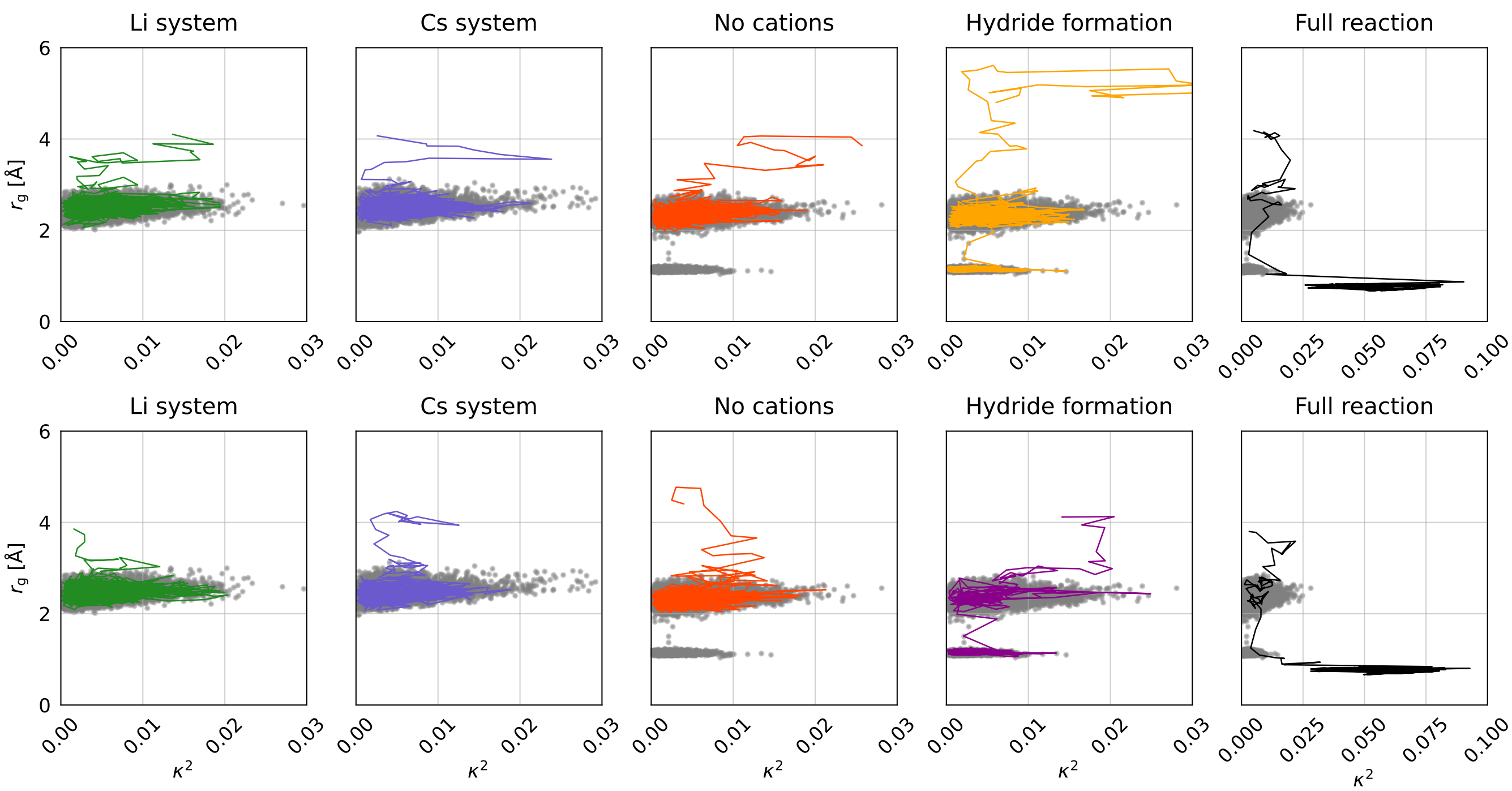

FIG. S9. Two-dimensional distributions of the gyration radius r_g and relative shape anisotropy κ^2 for different cases: the non-reactive trajectories with Li^+, Cs^+, no cations, the two reactive trajectories leading to hydride formation, and the two trajectories leading to the formation of a hydrogen molecule.

S6. SUMMARY OF REACTIVE EVENTS

Setup	Systems	Length	Total Time	Reactivity
revPBE38–CP2K	Li cations	10 × 10 ps	100 ps	no reactions
	Cs cations	10 × 10 ps	100 ps	no reactions
	No cations	10 × 10 ps<br>20 × 5 ps	200 ps	rare reactions
revPBE38–VASP	Li cations	50 × 5 ps	250 ps	no reactions
revPBE0–CP2K	Li cations	5 × 3 ps	15 ps	no reactions
revPBE0–VASP	Li cations	5 × 5 ps	25 ps	no reactions

TABLE S1. Summary of simulated systems and observed reactivity.

S7. EXAMPLE OF SOLVATION DYNAMICS

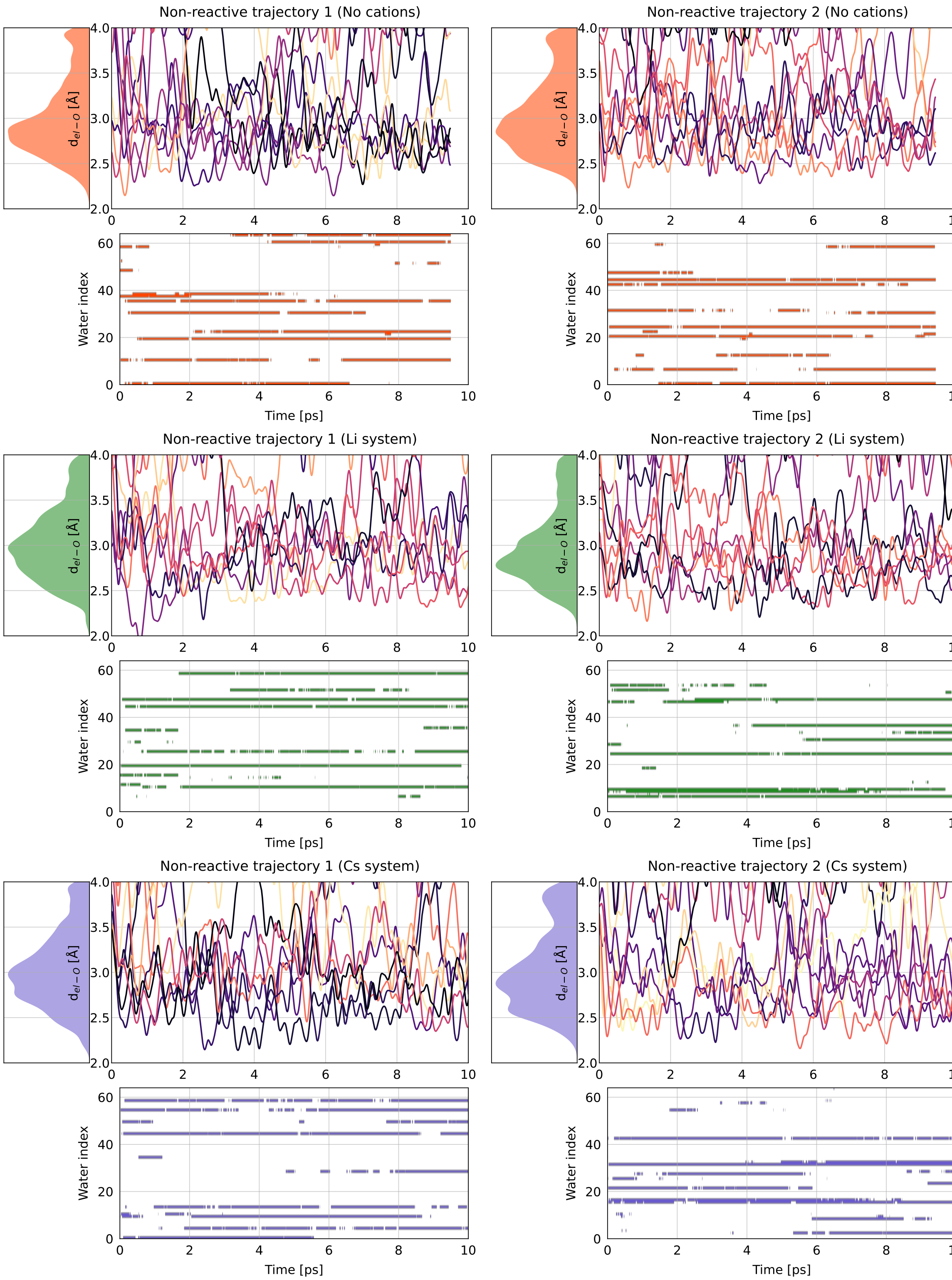

FIG. S10. Example of solvation dynamics of two non-reactive trajectories for each system (no cations, Li^+ and Cs^+). Each top panel: time evolution of the dielectron–oxygen distances within 4 Å from the dielectron center (smoothed using a Gaussian filter with $\sigma = 10$). Each side panel shows the distribution of these dielectron–oxygen distances. Each bottom panel: indices of water molecules within 3.6 Å illustrating the composition of the first solvation shell for the dielectron.
